# Acupoint catgut embedding improves senescence in a rat model of ageing by regulating mitophagy via the PINK1 pathway

**DOI:** 10.1111/jcmm.16295

**Published:** 2021-03-01

**Authors:** Meiling Zhou, Yumei Yuan, Zishu Lin, Bin Zhang, Wenluo Qin, Yongqiang Liu, Cong Yang, Qi Wang, Hong Zhang

**Affiliations:** ^1^ Department of Traditional Chinese Medicine The First Affiliated Hospital Sun Yat‐Sen University Guangzhou China; ^2^ Department of Rehabilitation Shenzhen Bao’An Hospital of Chinese Medicine Shenzhen China; ^3^ Acupuncture & Rehabilitation Clinical Medicine College Guangzhou University of Chinese Medicine Guangzhou China; ^4^ Department of Acupuncture and Moxibustion The First Affiliated Hospital of Guangzhou University of Chinese Medicine Guangzhou China; ^5^ Science and Technology Innovation Center Guangzhou University of Chinese Medicine Guangzhou China; ^6^ Institute of Clinical Pharmacology Guangzhou University of Chinese Medicine Guangzhou China

**Keywords:** acupoint catgut embedding, ageing, mitochondria, mitophagy, PINK1

## Abstract

Ageing population is a tough task worldwide, and the aggravating trend of ageing population in China brings enormous pressure to healthcare system. Chinese acupuncture has shown definite anti‐ageing effect as arthralgia relief, movement improvement, energy increase and immunity enhancement; however, the mechanisms underlying are far away from illumination. Increasing literature has highlighted the role of alterations in mitochondrial function as a potential central regulator in ageing biology; mitophagy plays a critical role in mitochondrial quality control. In the present study, we demonstrated that acupoint catgut embedding treatment ameliorated ageing‐related alterations in appearance, muscle function and spatial memory in rats, reduced degenerated cells in hippocampus, and maintained relatively normal structures in the hippocampus tissue and neurons. These changes were proved to be associated with the regulation of mitochondrial function and autophagic activity. Furthermore, we investigated part of the molecular mechanisms and demonstrated that the PINK1 other than PINK1–Parkin signalling pathway involved in the effects of acupoint catgut embedding, and the imbalancement between mitochondrial fusion and fission and stimulation of mitochondrial biogenesis may aggravate or compensate for impaired mitochondria. The factors act downstream PINK, and the interaction between them for mitochondrial homeostasis in this process remains to be identified.

## 1 INTRODUCTION

China is encountering aggravating trend of ageing. It is predicted that by 2050 there will be 400 million aged 65+ (26.9% of the total population), and 150 million aged 80+,^1^, ^2^, ^3^ one consequence brings about by the substantial demographic change is an explosure in the prevalence and incidence of age‐associated diseases, bringing enormous challenge into the healthcare system, the prevention and early intervention of these diseases is of great significance to protect the public healthcare resources from exhaustion. Chinese acupuncture contributes to ‘health aging’ with its definite effect as arthralgia relief, movement improvement, energy enhancement, while the mechanism underline remains much to explore, accumulating research highlighted the role of alterations in mitochondrial function as a potential action site in progress of acupuncture in stroke, Alzheimer's disease and vascular dementia. As cell's ‘power plants’, mitochondria play a vital role in energy production, cell senescence, stem cell functions, inflammatory responses, nuclear gene expression, epigenetics, Ca^2+^ homeostasis, apoptosis, telomere maintenance and gene integrity.^4^, ^5^, ^6^ Its dysfunction contributes to cell ageing and cell death, and has been regarded as a hallmark of senescence.^7^, ^8^ It would probably exist a link between mitochondria and acupuncture in the process of ageing improvement effect, besides, our early work demonstrated that autophagy was involved in acupuncture's anti‐ageing effect^9^; we therefore explored further whether mitophagy, the turnover of mitochondria, took part in action on ageing by acupuncture.

To explore these mechanisms, we mimicked human ageing in rats with a compound ageing model and intervened with acupoint catgut embedding, a special acupuncture that gains continuously stimulation in acupoints. Instead of stimulating acupoint with needles and removing immediately, acupoint catgut embedding is operated with absorbable surgical suture, embedding and detaining in points, with effect prolonging till sutures being absorbed and degraded, which could achieve similar effect without repeated operation per day to minimize operative interference. Emerging literature indicated that acupoint catgut embedding could benefit people with abdominal obesity, insomnia and diabetic gastroparesis,^10^, ^11^, ^12^ and improves dysmenorrhoea in primary dysmenorrhoea rats by inhibiting the activation of NLRP3 inflammasome in the uterine tissue,^13^ improve changes of structure of hippocampal neurons in senile chronic stress rats.^14^ It appeared attractive to figure out whether acupoint catgut embedding could also act on pathological process during senescence and whether or not mitochondria or mitophagy were involved in this process.

## 2 MATERIALS AND METHODS

### 2.1 Materials

In the experiments, we used the following reagents: d‐galactose (G0625‐100G) and dihydroethidium (DHE) dye solution were purchased from Sigma‐Aldrich; rapamycin (R817296) was obtained from Macklin Biochemical Technology; 3‐methyladenine (3‐MA; HY‐19312) was obtained from MedChemExpress; LC3‐double‐fluorescent lentivirus RFP‐GFP IRES‐puromycin was provided by GeneChem Co. Ltd.; and the absorbable surgical suture (3‐0, chromic) was obtained from Shanghai Pudong Jinhuan Medical Products Co. Ltd.

The following reagent kits were used: the senescence β‐galactosidase staining kit, Nissl staining solution, adenosine triphosphate (ATP) assay kit and 4′,6‐diamidine‐2′‐phenylindole dihydrochloride (DAPI) were provided by Beyotime Biotechnology; 2.5% glutaraldehyde (electron microscope fixative fluid) was obtained from BaiAoLaiBo Biotechnology; the mitochondrial protein extraction kit was obtained from Nanjing Jiancheng Biocompany; the mitochondrial membrane potential detection kit was provided by KeyGen Biotech; the bicinchoninic acid (BCA) protein quantitative kit was obtained from Sigma‐Aldrich; and the ECL chemiluminescence kit was provided by Beyotime Biotechnology.

The following primary antibodies were used: anti‐GAPDH (1:1000; Bioss Antibodies, bs‐10900R); anti‐LC3B (1:2000; Abcam, ab192890); anti‐p62 (1:10 000; Abcam, ab109012); anti‐Parkin (1:1000; Abcam, ab77924); anti‐MFN2 (1:5000; Abcam, ab124773); anti‐DRP1 (1:1000; Abcam, ab184247); and anti‐PGC1α (1:1000; Abcam, ab188102). HRP‐conjugated secondary antibodies were as follows: goat anti‐rabbit IgG H&L (1:10 000; Abcam, ab205718) and horse antimouse IgG H&L (1:5000; Cell Signaling Technology, 7076s).

### 2.2 Animal model and drug treatment

Young male‐specific‐pathogen‐free (SPF) Sprague‐Dawley rats (108‐220 g) were purchased from the laboratory animal centre of Guangzhou University of TCM (Guangzhou, China) aged 8 weeks and were allowed to adapt to the local housing facility for 1 week before the experiment began. All animal procedures were approved by the laboratory animals ethical review committee of Guangzhou University of TCM, in accordance with Chinese law for animal protection and the relative directive (Certificate No. 20181113040). Animals were randomly divided into six groups (n = 15 per group): the control group, model group, acupoint catgut embedding group, sham catgut embedding group, rapamycin group and 3‐MA group. Rats in the control group were given intraperitoneal injections of saline for 10 weeks (5 mL/kg/d), while the compound ageing model was induced to rats in the rest groups by intraperitoneal injections of d‐galactose solution (30 mg/mL, 5 mL/kg/d) plus chronic unpredictable stress. Animals in the acupoint catgut embedding group were given catgut embedding once a week. For catgut embedding, after anaesthesia, aseptic absorbable surgical sutures (1 cm per segment) were embedded at acupoints with an injection needle. The acupoint prescription is Baihui (GV20), Zusanli (ST36), Hegu (LI4) and Taichong (R3) divided into two groups: Baihui and Zusanli were one group, Hegu and Taichong were the other group. Each group of acupoints was alternative stimulated once a week. Animals in the sham acupoint catgut embedding group were given sham operations of catgut embedding, after anaesthesia, the same acupoints as catgut embedding group were stimulated with unloaded injection needle, with no material remaining in tissues, the prescription of acupoints selection and stimulation frequency was same as in acupoint catgut embedding group. Animals in the rapamycin group and 3‐MA group were pre‐implanted with a microdosing cannula (RWD) connecting to lateral ventricle, among them, animals in rapamycin group were administered rapamycin (1.5 nmol/5 µL, 5.49 µg/kg) via microinjection into the lateral ventricle once a day for 2 weeks in addition to treatment as the model group, animals in the 3‐MA group were given intraventricular microinjection of 3‐MA (400 nmol/5 µL) daily for 2 weeks in addition to the same treatment as the acupoint catgut embedding group. All animals were handled as described above for 10 weeks. Of the 15 rats in each group, 10 rats were killed 24 hours after behavioural experiments were finished. The remaining five rats per group were injected with LC3‐double‐fluorescent lentivirus in the hippocampus region (5 µL/hemisphere) and killed 14 days after the injection.

### 2.3 Microinjection and fluorescence detection of LC3‐double‐fluorescent lentivirus

Rats were anaesthetized and fastened to a brain stereotaxic apparatus (RWD Life Science, 68002) with their heads horizontal. After incising the skin and opening the skull, the hippocampus position was confirmed and drilled into (Bregma AP: −3.4, ML: −1.7, DV: −2.7). A microsyringe containing 3 µL of lentivirus was lowered at a speed of 1 mm/min into the hippocampus and injected at a constant speed of 0.5 µL/min. After the injection was given, the microsyringe was kept in place for 5‐10 minutes. Rats were sutured and kept warm before being allowed to wake up, and were killed 2 weeks after surgery. After a graded dehydration in sucrose, brain tissue was frozen and the hippocampus was sectioned at 30 µm, the nuclei were stained with DAPI, and sections were then observed using a laser scanning confocal microscope.

### 2.4 Motor coordination measured by the rotarod test

The rotarod test was carried out for 3 days. On the first 2 days, animals were trained on the rotarod at a speed of 16 rpm for three 5‐minute periods, with a 30‐minute break between each period. The falling latency and the number of times that falling occurred within 5 minutes were collected, and the averages over the three training sessions were then calculated. On day 3, the same protocol was used, but only the falling latency was recorded. The average value of the three rotarod sessions was calculated.

### 2.5 Spatial learning and memory ability estimated by the Morris water maze

The Morris water maze consisted of 5 days of orientation navigation tests and a 1‐day spatial probe test. During the orientation navigation tests, the platform was positioned at the centre of a quadrant, 1.5 cm underwater, and the animals were placed in the water from three to four entry points and allowed to explore the maze for 90 seconds. The time spent searching for the escape platform (the escape latency) was recorded. The training session was repeated for 5 days. In the spatial probe test, the underwater platform was removed, and rats were placed in the water at the R point. The number of times that the animals entered the platform quadrant and crossed the original platform within 90 seconds was collected and analysed.

### 2.6 Senescence assessment measured by β‐galactosidase staining

Hippocampal senescence was detected using a β‐galactosidase staining kit. The working liquid was prepared as follows: staining fluid C 930 µL, staining fluid A 10 µL, staining fluid B 10 µL and X‐Gal fluid 50 µL were sequentially added and mixed. Fresh frozen sections were air dried, fixed with the β‐galactosidase fixative solution for 15 minutes and washed three times with distilled water. To stain the sections, several drops of working liquid were placed upon each section and incubated at 37°C for 16‐18 hours, before being washed three times with distilled water and coverslipped with glycerine jelly.

### 2.7 Hippocampal pathomorphology by Nissl staining

To investigate pathomorphology in the hippocampus, sections were stained with Nissl staining solution. After being paraffin embedded and sectioned at 30 µm, the brain tissue was deparaffinized and rehydrated in the following reagents: three times in xylene for 10 minutes, absolute ethanol for 5 minutes, 90% ethanol for 2 minutes, 70% ethanol for 2 minutes and distilled water for 2 minutes. For the Nissl staining, sections were incubated in Nissl staining solution for 10 minutes at 50°C and then washed twice in distilled water. Sections were then placed in 95% ethanol for 5 seconds and then in 70% ethanol two times. Finally, the sections were dehydrated twice in 95% ethanol for 2 minutes, cleared in xylene twice for 5 minutes and coverslipped with neutral gum.

### 2.8 Electron microscopy

Rats were anaesthetized and perfused completely with normal saline and paraformaldehyde. Next, the CA1 region of the hippocampus was isolated and four or five specimens (approximately 1 mm × 1 mm × 1 mm) were taken and fixed in 2.5% glutaraldehyde solution away from light for 3‐5 days at 4°C, and then scanned with a transmission electron microscope.

### 2.9 Detection of ATP

Hippocampal ATP was detected using an ATP assay kit. Once the dyeing incubator was prepared, sections were incubated in the incubation solution at 37°C for 30 minutes, washed with distilled water for 2 minutes, treated with ammonium sulphide for 1 minute and washed with running water for 5 minutes. Under a microscope, the ATP‐positive cells were brown‐black at the site of ATP activity.

### 2.10 Detection of reactive oxygen species (ROS)

Hippocampal ROS was detected using DHE dye solution. Frozen sections of fresh tissue were refrigerated, circled with an immunohistochemical pen and incubated in DHE dye solution at 37°C for 30 minutes, before undergoing three 5 minutes washes in PBS (pH 7.4). Slices were dried slightly and then stained with DAPI solution at room temperature for 10 minutes. After washing the sections three times in PBS and drying them slightly, the sections were coverslipped with an anti‐fluorescence quenching agent.

### 2.11 Detection of the mitochondrial membrane potential

Mitochondrial membrane potential was tested using a mitochondrial protein extraction kit, a mitochondrial membrane potential detection kit and a BCA protein quantitative kit. Fresh hippocampus weighing approximately 150 mg was cut into small pieces, added to 1.5 mL of pyrolysis solution and homogenized at low speed in an ice bath. The solution was then centrifuged at 800 *g* at 4°C for 5 minutes, and the supernatant was acquired and slowly injected along the centrifuge tube wall to cover the upper layer of liquid A. This solution was then centrifuged at 15 000 *g* at 4°C for 10 minutes, and the supernatant was discarded; the remaining precipitate was mitochondria. To measure mitochondrial membrane potential, the purified mitochondria were first quantified using the BCA protein quantitative kit. The concentration of each group was adjusted to 3 mg/mL, and the mitochondria were then resuspended in 1× assay buffer. The JC‐1 working fluid was made up of 1× assay buffer and JC‐1 at a ratio of 500:1. The JC‐1 working fluid and the mitochondrial solution were then added to a 96‐well plate at a ratio of 9:1, and fluorescence changes were observed with a fluorescence microscope.

### 2.12 Immunoblotting

Hippocampal tissue was treated with radio immunoprecipitation assay (RIPA) buffer and phenylmethanesulfonyl fluoride (PMSF), homogenized into a uniform suspension and centrifuged at 4°C for 15 minutes. The extracted proteins were then quantified using the BCA kit; 20‐25 µg of proteins from each group was loaded onto a 12% SDS‐PAGE gel, and after electrophoresis, the proteins were separated and transferred onto PVDF membranes. Non‐phosphorylated proteins were blocked by 7% non‐fat milk at room temperature for 2 hours and washed with TBST. Next, the protein‐loading membranes were incubated overnight at 4°C with the following primary antibodies: anti‐GAPDH (1:1000), anti‐LC3B (1:2000), anti‐p62 (1:10 000), anti‐Parkin (1:1000), anti‐MFN2 (1:5000), anti‐DRP1 (1:1000) and anti‐PGC1α (1:1000). After being washed with TBST, the membranes were incubated with the corresponding secondary antibody for 1 hour, and protein expression was detected using an ECL chemiluminescence kit. Membranes were then scanned with a ChemiDoc XRS (Bio‐Rad), and the resulting bands were quantified using Image Lab software v3.0.1 (Bio‐Rad).

### 2.13 Statistical analysis

Statistical analysis was performed using Stata v17.0 software (Stata Labs). If the data conformed to normal distributions, they were presented as mean ± SEM, and comparisons among groups were performed using a one‐way analysis of variance (ANOVA). If the data did not conform to normal distributions, they were presented as median p50 with rank sum test, and multigroup comparisons were carried out using a Kruskal‐Wallis test. *P* values < .05 were considered significant, and all results were verified by using at least three independent experiments.

## 3 RESULTS

### 3.1 Acupoint catgut embedding improves senescent state and behaviour in ageing rats

**Figure 1:**
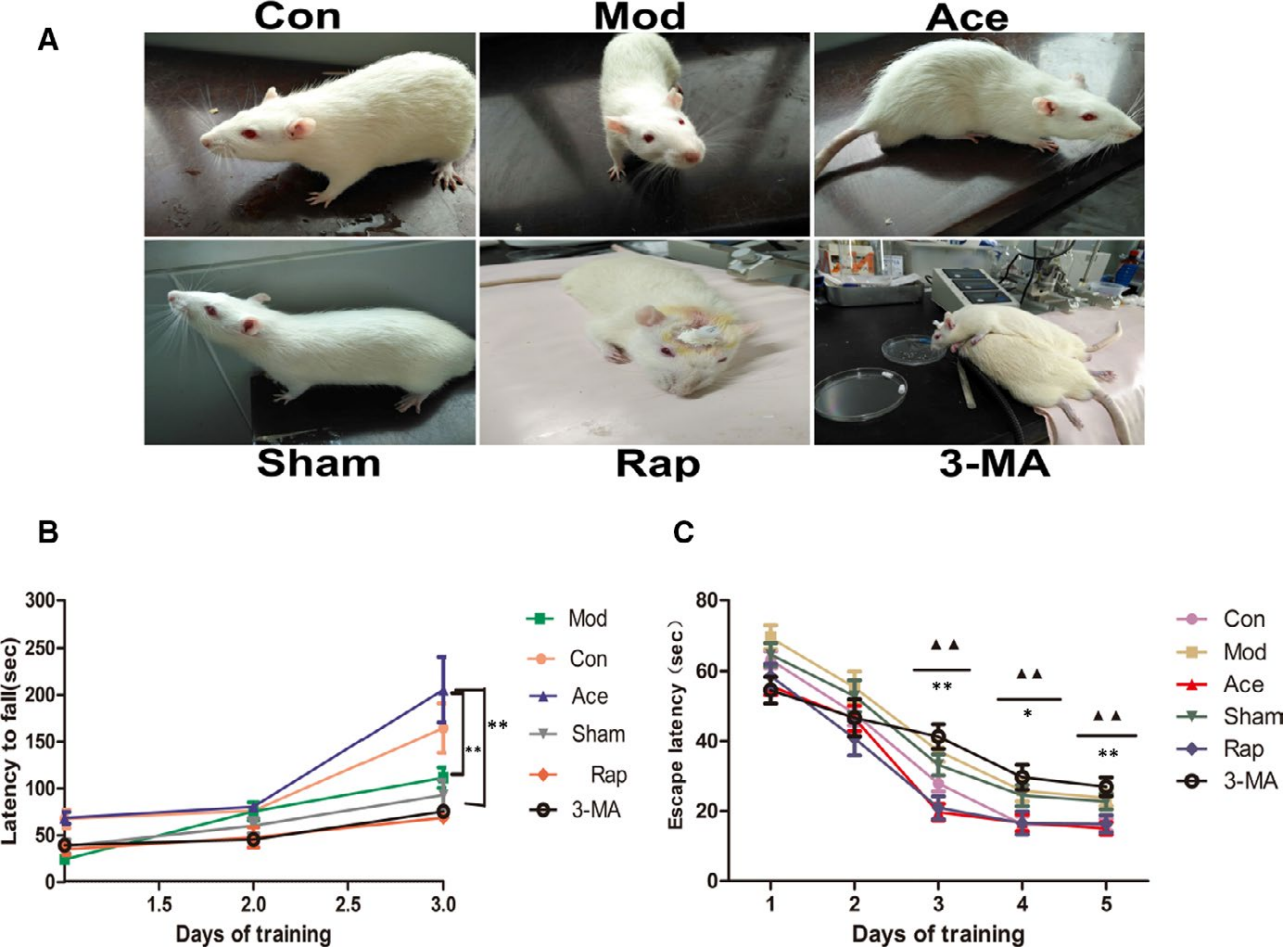
Acupoint catgut embedding improves senescent state and behaviour in ageing rats. A, The appearance of each group after the operation. B, The rotarod test. Rotarod performance of control (n = 10), model (n = 11), Ace (acupoint catgut embedding group; n = 11), sham (sham catgut embedding group; n = 9), Rap (rapamycin group; n = 11) and 3‐MA (3‐methyladenine group; n = 10) rats. Retention time on the rotating rod at 16 rpm was measured, and data are expressed as mean ± SEM. There were significant differences between mod and Ace, Ace and 3‐MA **P < .01 at the third day of training, while no significant differences shown between Con and Mod, Mod and Sham, Mod and Rap, Ace and Sham, Ace and Rap (*P = *ns). C, Learning curves from the Morris water maze. There were significant differences in learning between mod and Ace **P < .01, *P < .05; and between Ace and 3‐MA ^▲▲^ *P* < .01, no significant variation shown between Con and Mod, Mod and Sham, Mod and Rap, Ace and Sham, Ace and Rap (*P = *ns)

We first observed whether acupoint catgut embedding could improve the ageing state in rats. As shown in Figure 1A, in the rat model of ageing (the model group), animals were noted to have emaciated bodies, messy and withered fur, pale pinna, dim eyes, lower appetite, reduced activity and phlegm in the throat, and showed initial irritation when handled before becoming unresponsive. In contrast, acupoint catgut embedding treatment in the ageing rat model (the acupoint catgut embedding group) caused a significant improvement in the manifestation of ageing, and animals were noted to have well‐proportioned bodies, bright and smooth fur, ruddy auricles and claws, a normal diet, and flexible responses when being handled (Figure 1A). We were also interested in whether acupoint catgut embedding affected locomotion and memory ability in ageing rats; thus, we also measured motor coordination using the rotarod test. Rats in the acupoint catgut embedding group spent longer on the rotating rod compared with rats in the model group, indicating that acupoint catgut embedding, which gives continuous stimulation to certain acupoints, may benefit motor coordination in ageing rats (Figure 1B). In addition, we investigated spatial learning and memory ability using the Morris water maze. In this test, we observed that rats in the acupoint catgut embedding group had shorter escape latency times from day 3 of training onward compared with rats in the model group, indicating that acupoint catgut embedding may improve spatial learning and memory ability in ageing rats (Figure 1C). Additionally, we investigated whether autophagy might play a role in this process by observing rats that were also treated with the autophagy promoter rapamycin (the rapamycin group) or the autophagy inhibitor 3‐MA (the 3‐MA group). Rats in the rapamycin group also had a shorter escape latency compared with rats in the model group, while rats in the 3‐MA group had a prolonged escape latency compared with the acupoint catgut embedding group (Figure 1C). These results indicate that the inhibition of autophagy reduced the memory improvement effect of acupoint catgut embedding treatment, suggesting that autophagy has at least some effect on learning and memory impairment during ageing.

### 3.2 Acupoint catgut embedding ameliorates hippocampal ageing in rats

**Figure 2:**
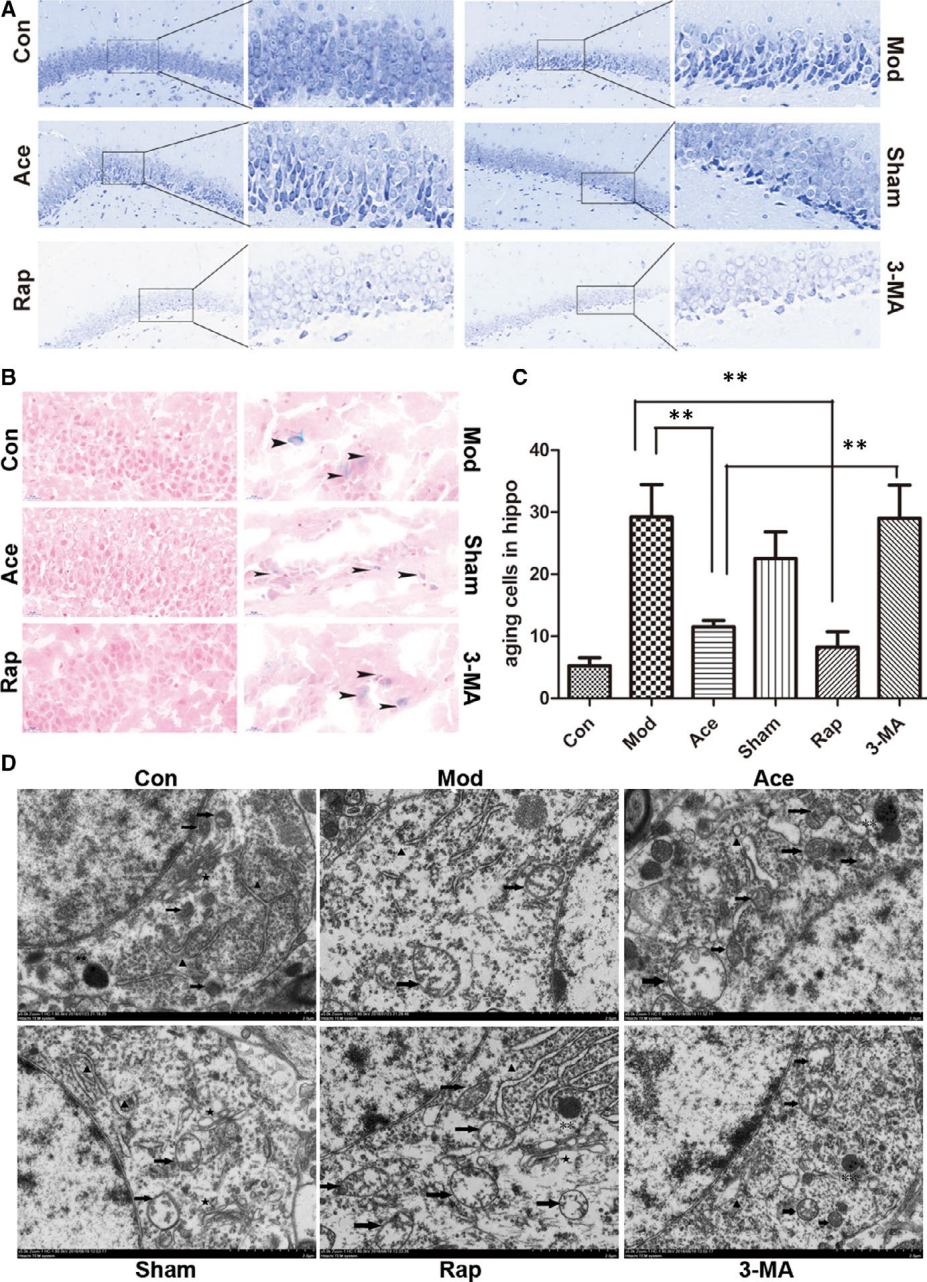
*A*cupoint catgut embedding ameliorates hippocampal ageing in rats. A, Hippocampal pathological manifestations with Nissl staining (200×, 600×): In the control group, neurons were arranged in an orderly fashion and were round and lightly stained, with Nissl bodies evenly distributed in axon hillocks. In the model group, many shrunken and deformed cells were observed in the dentate gyrus (DG). With acupoint catgut embedding treatment, hippocampal neurons were neatly arrayed, with many approximately normal cells and diffuse degenerated cells in the DG. In the sham catgut embedding group, deformed, strongly stained cells were observed in the DG. In the rapamycin group, neurons were arranged in an orderly fashion, and there were few hyperchromatic neurons. In the 3‐MA group, scattered hyperchromatic neurons were observed in the DG. B and C, Hippocampal senescence using β‐galactosidase staining (500×). The black arrows indicate ageing cells. In the control group, hippocampal neurons were orderly arranged without any obvious ageing cells, while in the model group, there were widely scattered blue‐stained ageing cells. In the acupoint catgut embedding group, there was a marked reduction in ageing cells in the hippocampus compared with the model group (*P* < .01). In the rapamycin group, similar results were seen as in the acupoint catgut embedding group (*P* < .01), while 3‐MA treatment partly blocked the anti‐ageing effect of acupoint catgut embedding (*P* < .01). No significant variation seen between the model group and the sham acupoint catgut embedding group (*P = *ns). D, Endoplasm ultrastructure in hippocampal neurons using transmission electron microscopy (5000×; → indicate mitochondria, ^▲^ indicate the endoplasmic reticulum, ** indicate lysosomes, and ^★^ indicate Golgi apparatus. In the model group, the endoplasm of neurons was sparse and contained degenerated organelles, and mitochondria had reduced, swollen and fractured cristae. We also observed degranulated endoplasmic reticulum, accumulations of lipofuscin, and chromatin agglutination and marginalization. Acupoint catgut embedding treatment improved the neuronal endoplasm, with relatively normal mitochondria and all stages of lysosomes observed. Ultrastructure in the sham group showed no apparent difference from that of the model group. In neurons of the rapamycin group, organelles were regularly arranged with many swollen mitochondria, endoplasmic reticulum and lysosomes, while in the 3‐MA group, the neuronal endoplasm was similar to that of the model group

After observing that acupoint catgut embedding in rats delayed the signs of ageing in the hair, diet, spontaneous activity, mental state, motor coordination, and learning and memory ability, we further investigated whether acupoint catgut embedding also plays a role in ageing in the hippocampus, which is the brain area related to spatial learning and memory. Nissl staining revealed that acupoint catgut embedding modified hippocampal ageing; there were neatly arrayed, relatively normal‐shaped neurons and diffuse degenerated cells in the dentate nucleus (DG) of rats in the acupoint catgut embedding group, compared with the shrinkage and deformed cells that were observed in the DG of model group rats. The application of 3‐MA attenuated the effects of acupoint catgut embedding on hippocampal cell morphology (Figure 2A). β‐galactosidase staining further confirmed that acupoint catgut embedding in ageing rats markedly reduced ageing cells in the hippocampus compared with the model group and that 3‐MA was able to partly block this anti‐ageing effect (Figure 2B,C). Furthermore, cell ageing is generally characterized by organelle dysfunction and degeneration; thus, transmission electron microscopy was used to observe ultrastructural changes in hippocampal neurons. Neurons in the model group were observed to be degenerated, with disarrayed organelles and mitochondria that had reduced, swollen and fractured cristae. In addition, we noted degranulation of the endoplasmic reticulum, sparse synapses, collapsed cytoskeletons, and accumulation of lipofuscin in the model group. In contrast, in the acupoint catgut embedding group, we observed only slightly swollen mitochondria, along with a few lysosomes at different stages, while neurons in the 3‐MA group were similar to those of the model group (Figure 2D). At the level of both tissue and cells, a modified manifestation of hippocampal ageing was observed with acupoint catgut embedding treatment.

### 3.3 Acupoint catgut embedding partly enhances mitochondrial function in cell ageing

**Figure 3:**
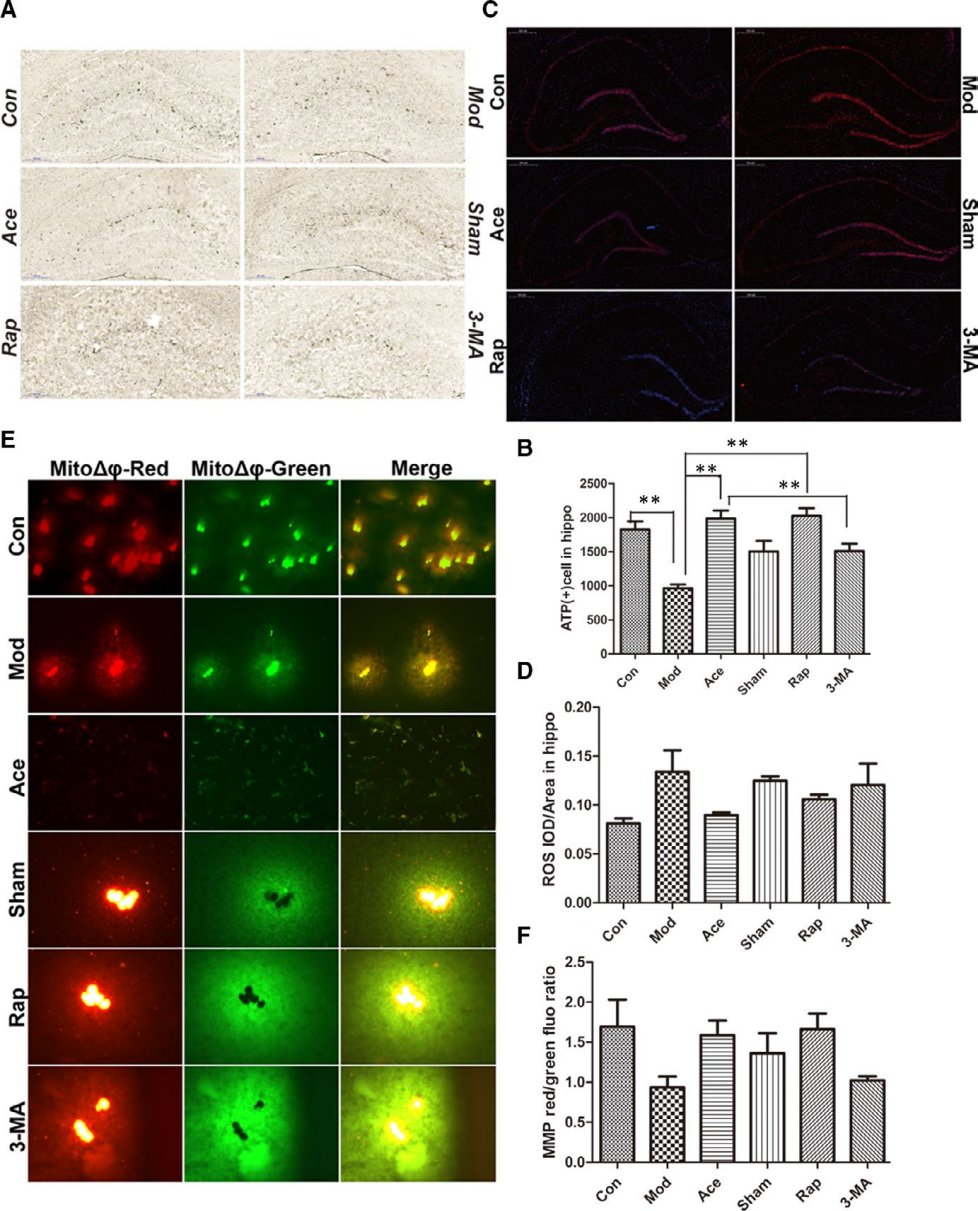
Acupoint catgut embedding partly enhances mitochondrial function in cell ageing. A and B, Hippocampal ATP production was quantified by counting the number of cells stained black. ATP(+) cells (35×; mean ± SEM; from three independent experiments; **P < .01). There was a significant reduction in hippocampal ATP production in the model group compared with the control group. Acupoint catgut embedding alleviated this decline, while the addition of 3‐MA offset the alleviating effect of acupoint catgut embedding treatment (*P* < .01), there was no significant difference between the model group and sham acupoint catgut embedding (*P = *ns). C and D, Hippocampal ROS expression was quantified by mean optical density (integrated density/area) of red fluorescence (30×; mean ± SEM; from three independent experiments). In the model group, ROS generation demonstrated a slightly up‐regulated tendency but not yet reached statistical difference, when compared with the control group (*P = *ns), with intense red fluorescence in the hippocampus. Acupoint catgut embedding partial slowed this upward trend as compared to the model group with no significant difference, (*P = *ns), while autophagic inhibition with 3‐MA partly reversed the effect of acupoint catgut embedding treatment (with no significant difference, *P = *ns). E and F, Mitochondrial membrane potential was represented by the ratio of red fluorescence to green fluorescence. Healthy mitochondria mainly reflected red fluoresce, while injured mitochondria reflected green fluoresce (200×; mean ± SEM; from three independent experiments). Mitochondria from the model group appeared to have compromised mitochondrial transmembrane potential compared with the control group (*P = *ns). Acupoint catgut embedding treatment may partial ameliorate membrane potential depolarization without statistical difference (*P = *ns), while the addition of 3‐MA to some extend blocked the effect by acupoint catgut embedding treatment (*P = *ns)

We next detected whether mitochondrial dysfunction is involved in the manifestations of cell ageing because we observed that neuronal senescence was accompanied by increased degeneration in organelle structure, with swollen, fractured cristae observed in the mitochondria. Mitochondria provide most of the energy in a cell, but they also produce ROS as a by‐product of this energy production. Mitochondrial dysfunction often manifests as a decrease in the mitochondrial membrane potential. We therefore measured mitochondrial energy production using ATP dyeing in the hippocampus. In the model group, there was a significant reduction in ATP production compared with the control group, and ATP production was concentrated in the perineuronal tissue. Acupoint catgut embedding treatment alleviated this downward trend, and ATP was mainly produced in neuron‐intensive areas in this group. 3‐MA could offset the alleviating effect of acupoint catgut embedding treatment (Figure 3A,B). Moreover, we also estimated ROS production using fluorescent staining. Rats in the model group generated more ROS, with more intense fluorescence in the hippocampus compared with the control group. Acupoint catgut embedding slowed such an increase, while autophagic inhibition with 3‐MA partially inhibited the acupoint catgut embedding effect (Figure 3C,D). Additionally, mitochondrial membrane potentials were detected using the fluorescent probe JC‐1, and mitochondria had decreased mitochondrial transmembrane potentials in the model group compared with the control group. Acupoint catgut embedding treatment improved membrane potential depolarization, while 3‐MA treatment obstructed the improvement caused by acupoint catgut embedding (Figure 3E,F).

### 3.4 Acupoint catgut embedding mitigates autophagic decline in ageing rats

**Figure 4:**
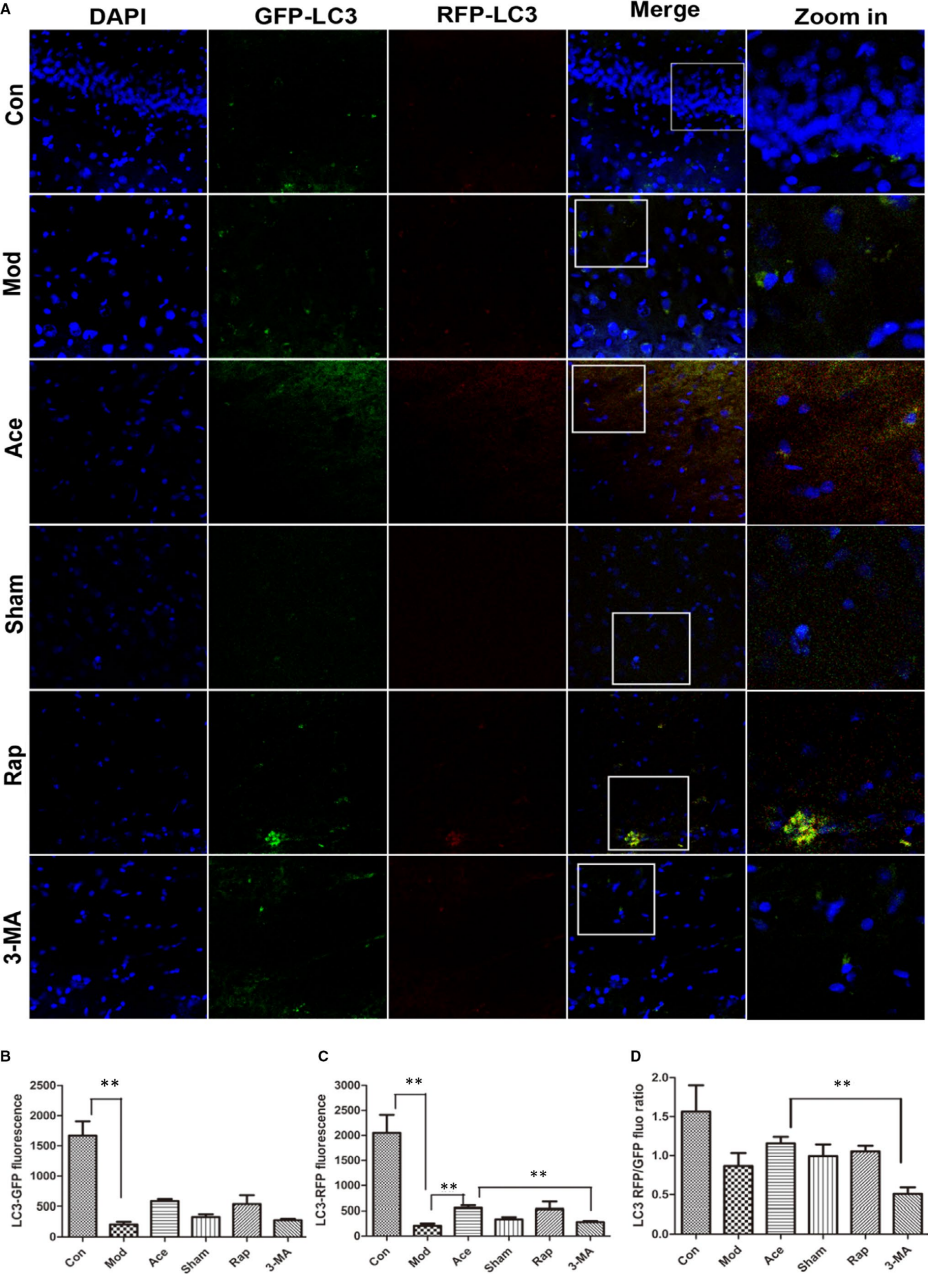
Acupoint catgut embedding mitigates autophagic decline in ageing rats. A–D, Autophagic activity in the hippocampus of the control, model, acupoint catgut embedding, sham acupoint catgut embedding, rapamycin and 3‐MA groups. Autophagic activity was quantified by LC3‐PAGFP fluorescence, and autophagy flow fluency was represented by the RFP/GFP ratio (400×; mean ± SEM; from three independent experiments; **P < .01). In the hippocampus of the model group, we observed a rapid decrease of LC3‐PAGFP fluorescence intensity (*P* < .01) and a lower RFP/GFP ratio compared with the control group (*P* = ns). Acupoint catgut embedding treatments were able to ease this downward trend in red fluorescence intensity (*P* < .01). 3‐MA treatment partial blocked the effects of acupoint catgut embedding concerning red fluorescence intensity and RFP/GFP ratio (*P* < .01), beyond that, no significant differences were seen between the model and the sham acupoint catgut embedding groups, the model and Rapamycin (*P = *ns)

Accumulation of injured mitochondria is the result of an imbalance between mitochondrial damage and mitochondrial repair or elimination, from the early‐stage research we have observed that there were many degenerated mitochondria in the cells of ageing rats, we were also interested in whether mitochondria clearance, or mitophagy, also contributes to ageing‐mediated mitochondrial degeneration. We therefore measured autophagic activity using intraventricular microinjections of LC3‐double‐fluorescent lentivirus (3 µL per hemisphere at 0.5 µL/min) to directly visualize and quantitatively measure autophagic activity through the expression of an LC3‐targeted photoactivatable acid‐sensitive GFP (PAGFP). As a PAGFP‐labelled autophagosome fuses with a lysosome, green fluorescence is quenched and red fluorescence is highlighted, and this partly represents autophagic flow fluency. In the model group, there was a rapid decrease of LC3‐PAGFP fluorescence intensity and a lower RFP/GFP ratio, indicating decreased autophagic ability compared with the control group. Rats in the acupoint catgut embedding or rapamycin groups had an easing of this trend towards a decrease, with a recovered RFP/GFP ratio, but this effect was eliminated by 3‐MA treatment (Figure 4A–D). These data suggest that a defective autophagic capacity is at least partly involved in ageing‐mediated mitochondrial degeneration. In addition, the results indicate that acupoint catgut embedding treatment provides a certain recovery effect, which is similar to rapamycin treatment but attenuated by 3‐MA treatment.

### 3.5 Acupoint catgut embedding delays ageing by regulating mitophagy via the PINK1–Parkin pathway

**Figure 5:**
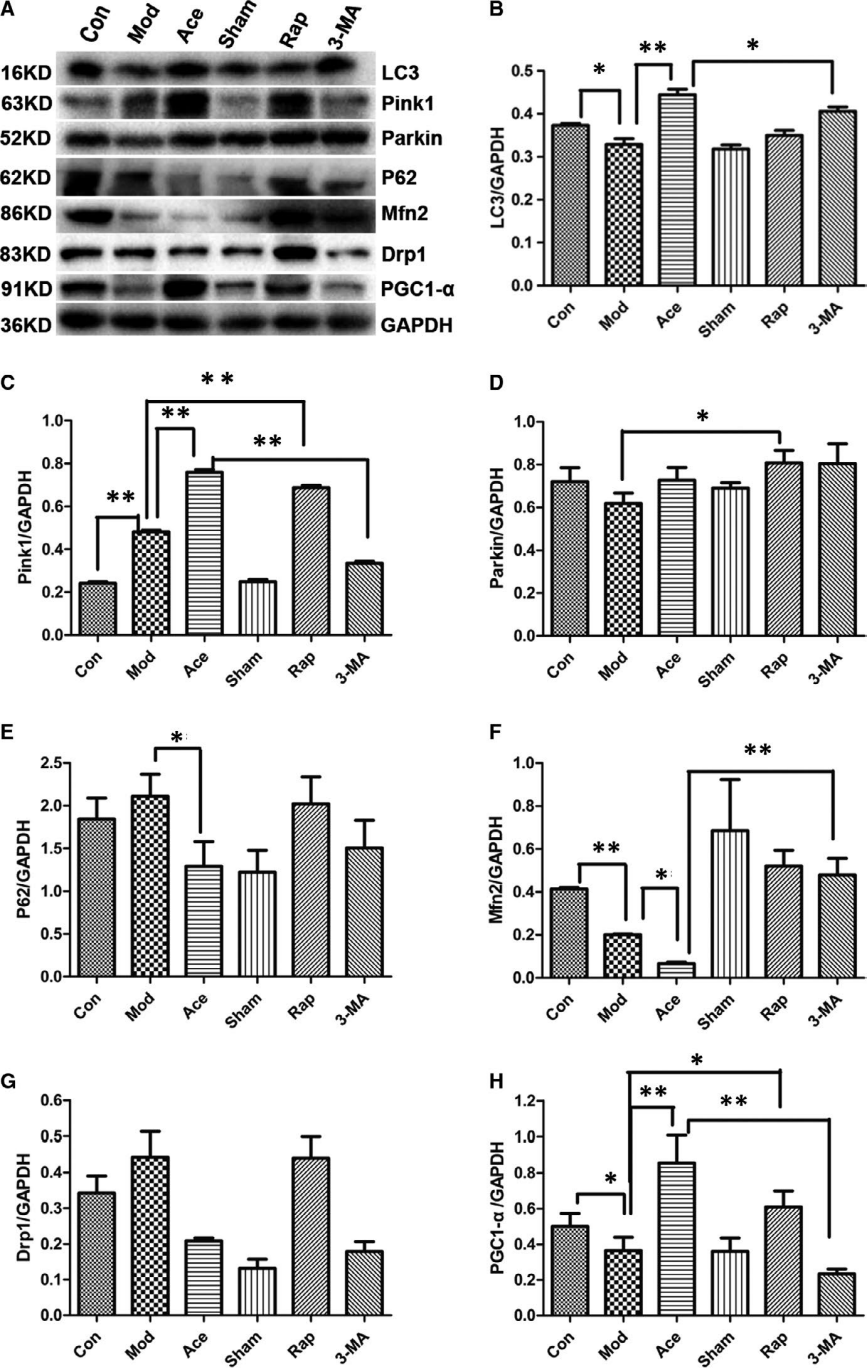
A–H, Mitophagy, mediated by the PINK1–Parkin pathway, and mitochondrial biogenesis are involved in ageing and in the ageing improvement effects of acupoint catgut embedding. The hippocampus from rats of the control, model, acupoint catgut embedding, sham acupoint catgut embedding, rapamycin and 3‐MA groups were prepared and analysed by Western blotting (mean ± SEM; from three independent experiments; **P < .01; *P < .05) (A) autophagy activity was measured by LC3 protein levels using an antibody against LC3B. Proteins in the PINK1–Parkin pathway were measured by PINK1, Parkin, P62, Mfn2 and Drp1 levels with relevant antibodies. Mitochondrial biogenesis was represented by PGC1‐α protein levels with an antibody against PGC1‐α. GAPDH was used as the loading control in all Western blots. Bar graphs show the levels of LC3 (B), PINK1 (C), Parkin (D), P62 (E), Mfn2 (F), Drp1 (G) and PGC1‐α (H). In the LC3 protein, rats in model group showed suppressed in expression as compared to control group (*P* < .05), acupoint catgut embedding treatment on ageing rats might reverse such downtrend in ageing process (*P* < .01), which may be cut down by 3‐MA (*P* < .05), while there was no statistic difference between the model rats and rats being treated by sham acupoint catgut embedding and by rapamycin (*P* = ns). As for Pink1 protein expression, an upward expression was seen in ageing rat model group compared to control group (*P* < .01), acupoint catgut embedding treatment and rapamycin on ageing rats could aggravate this upward trend (*P* < .01), whose effect could be inhibited by adoption of 3‐MA (*P* < .01), no significant difference showed between model group and sham acupoint catgut embedding treatment group (*P* = ns). As the expression of Parkin protein, the administration of rampamycin on ageing rats might boost its expression as compared to model group (*P* < .05), no significant difference showed between the rest groups (*P* = ns). In expression of P62 protein, rats in acupoint catgut embedding treatment group represented a reduction as compared to the model group (*P* < .05), and comparison of the rest groups showed no significant variation (*P* = ns). As for expression of Mfn2 protein, hippocampus in model group estimated a down‐regulation in expression when compared to the control group (*P* < .01), acupoint catgut embedding treatment on ageing rats might aggravate such trend (*P* < .05), which may be reversed by adding of 3‐MA (*P* < .01), while comparison between the model and sham treatment, model and rapamycin showed no significant difference (*P* = ns). In expression of Drp1 protein, there were merely upward inclination between control and model groups (*P* = ns), down‐regulation yet no statistic difference seen between the model group and acupoint catgut embedding treatment group and sham operation group (*P* = ns). In expression of PGC1‐α protein, a marked decrease showed between the control and model groups (*P* < .05), treatment with acupoint catgut embedding and/or rapamycin on ageing rats add extra expression as compared to ageing rats (*P* < .01, *P* < .05), this increased effect mediated by acupoint catgut embedding may be blocked by 3‐MA (*P* < .01), however, sham acupoint catgut embedding on ageing animals showed no remarkable influence in ageing rats as compared to rats in model group (*P* = ns)

After observing that there were many degenerated mitochondria in ageing rats and that defective autophagy was involved in this phenomenon, we next investigated whether mitophagy plays a role in ageing‐mediated mitochondrial degeneration. The PINK1–Parkin pathway is one of the classical pathways of mitophagy, with downstream substrates that include the mitochondrial fusion protein mitofusin2 (Mfn2) and the division protein Drp1, with the recognition of ubiquitinated substrates mediated by the autophagy receptor p62, for example. Increased damaged mitochondria are the result of an imbalance between mitochondrial clearance and generation; thus, we also detected whether defective mitochondrial biogenesis also contributed to the ageing‐mediated increase in degenerated mitochondria. We estimated protein expression levels using Western blots with hippocampal tissue. In the model group, there was decreased expression of LC3 compared with the control group. Acupoint catgut embedding reversed this downward trend, but this reversal was inhibited by the autophagy inhibitor 3‐MA (Figure 5A,B). In contrast to LC3, PINK1 expression was increased in the model group compared with the control group, and acupoint catgut embedding exacerbated this increase, although this exacerbation was inhibited by 3‐MA treatment. Parkin protein expression did not show any such trends (Figure 5A,C,D). For the mitophagy receptor protein p62, rats in the model group had higher expression than in the control group, while acupoint catgut embedding treatment caused a decline in this protein (Figure 5A,E). For Mfn2, a protein downstream of Parkin, there was reduced expression in the model group compared with the control group, and treatment with acupoint catgut embedding aggravated this decrease. Drp1 protein expression in the model group was not significantly different than in any of the other groups (Figure 5A,F,G). Finally, there was higher expression of PGC1‐α, a mitochondrial biogenesis protein, in the acupoint catgut embedding group compared with the model group, which may partly act as a compensatory response to mitochondrial injury.

## 4 DISCUSSION

Ageing is a subclinical pathological process characterized by a progressive decline, both physical and mental, in individuals. Recently, research on ageing delaying by traditional Chinese medicine springs up, while the mechanism underlying remains much to explore. Our current study revealed that the improvement in senescent rats by acupoint catgut embedding treatment was associated with regulation of mitophagy, manifested as improving senile appearance, ameliorating muscle coordination and endurance, slowing memory loss, preserving normal cellular morphology, reducing the ageing of cells in the hippocampal DG, ameliorating intracellular ATP and ROS generation, and preserving mitochondrial membrane potential decline in the hippocampus. These effects were similar to those of an autophagy promoter, and could be partial attenuated by the inhibition of autophagy. We also demonstrated that mitophagy effected by acupoint catgut embedding is associated with activation of the PINK1 signalling pathway.

The relation between mitochondria and ageing is complex which is far from clarified; emerging evidence has taken the alteration of mitochondrial function as a potential central regulator in the ageing process.^15^, ^16^, ^17^ Studies have reported that there are links between mitochondrial quality control and age‐dependent pathology, and mitochondrial‐to‐nuclear signalling might also regulate the rate of ageing.^12^ In the current study, we observed reduced mitochondrial mass and injured mitochondria with fractured cristae and vacuolar swelling. These degenerated mitochondria accumulated in the cytoplasm of ageing rats (Figure 2D). There was also an alteration in mitochondrial function, with defective energy production, accumulated ROS, (Figure 3), which is consistent with the previous analysis as approximately 50% decline in mitochondrial respiratory capacity and ATP generation in old patients compared with younger patients.^18^, ^19^ As it is described that mitochondrial damage is commonly accompanied by a decrease in mitochondrial membrane potential,^20^ these phenomena were also observed from our work in the ageing process (Figure 3). Acupoint catgut embedding acted on ageing rats by preserving normal mitochondrial structure, ameliorating ATP and ROS production, and maintaining mitochondrial membrane potential homeostasis. This indicates that mitochondria, as one of the target organ of acupoint catgut embedding, play a critical role in ageing improvement process, which is also revealed in the pathological progresses of insulin resistance, focal ischaemic stroke and spinal cord injury.^21^, ^22^, ^23^

As we have figured out that mitochondria played a role in the effect of acupoint catgut embedding on ageing process, it occurred to us that how could it act on ageing by mediating mitochondria? Or is there any pathway through which we could limit mitochondrial damage to slow down senescence process? Accumulating literature indicates that mitochondrial quality control is associated with age‐dependent pathology, mitophagy, as one of important part of—mitochondrial quality control, is reported to markedly decline in mammalian tissues during normal ageing^24^, ^25^; therefore, we made a hypothesis that dysfunction of mitophagy may involved in the cytoplasmic accumulation of defective mitochondria in ageing rats (Figure 2D). To further investigate the hypothesis, we used LC3‐double‐fluorescent lentivirus to inject into the hippocampus region (5 µL/hemisphere) and killed 14 days after injection. Autophagic activity alteration in age‐related pathology can be observed by a decreased expression of LC3 fluorescent protein in the hippocampus (Figure 4A,B), and it is also notable that the decline in fusion rate of autophagosome and lysosome cooperatively accounts for inefficiency of autophagic activity (Figure 4A,C). What is interesting in our work is the regional distribution of compromised autophagic activity, with concentrated LC3 fluorescence in CA2 region and relatively fewer fluorescence in DG region, which is consistent with distributed characteristic of hippocampus degenerated cell by nissl staining (Figure 2A), with shrunken and deformed cells centre at the dentate gyrus. It appears that there is a potential association between the regulation of mitophagy and ageing delaying, which is also reported that up‐regulating autophagy may delay the onset and ameliorate the symptoms of age‐related phenotypes.^26^ As we investigated further through which acupoint catgut embedding acts on mitochondria, we discovered that acupoint catgut embedding ameliorated the decline in autophagic activity in ageing process, which could be partial blocked by the autophagy inhibitor 3‐MA. Together, all these results pointed the fact that acupoint catgut embedding could improve ageing status at least partial by mediating autophagy and facilitating the elimination of defective mitochondria.

Mitophagy is reported to be involved in a variety of physiological process as fertilization, differentiation, development, age‐related disease prevention and health maintenance throughout life, the PINK1–Parkin signalling pathway is the most frequently studied by scientist. So it came up to us whether the PINK1–Parkin pathway took part in process of acupoint catgut embedding acts on mitochondria? We demonstrated that acupoint catgut embedding treatment on ageing animals was able to stimulate PINK1 expression in age‐related hippocampal tissues, but did not have direct effect on Parkin expression, suggesting that there may be alternative factors acting downstream of PINK1 in this process, such viewpoint is also raised by literature that PINK1 could recruit the autophagy receptor to the mitochondria in a PARK2‐independent manner, another research proved that PINK1 and BECN1 relocalize at mitochondria‐associated membranes during mitophagy and promote ER‐mitochondria tethering and autophagosome formation,^27^ which indicated that there was probably some undefined molecules that involved in this process. Similar trend also presented in p62 protein, whose expression was not consistent with the fluctuation of PINK1 or LC3 protein, either. It is likely that p62 protein, although represented as an important receptor for mitophagy in Parkinson's disease, is not necessary weighing equal when it transfer into pathologyduring ageing, in fact researches have gradually uncovered NIX/BNIP3L, BNIP3 and FUNDC1 as mitophagy receptors in mammalian system,^28^, ^29^, ^30^, ^31^, ^32^, ^33^, ^34^ which may explain insufficient interaction between p62 and autophagy activity regulation by acupoint catgut embedding here. Being substrates downstream of ubiquitinase as Parkin, Mfn2 and Drp1 also belong to critical members in mitochondrial dynamics family. Though involved in opposite direction as fusion and fission, Mfn2 and Drp1 did not exhibit contradictious relation as ‘one falls, another rises’ in this research, which may attribute to ageing‐related disruption of homeostasis between mitochondrial fusion and fission, such imbalance reversely aggravated the chaos within mitochondria and cells. Besides, an anabolic‐catabolic imbalance between mitophagy and mitochondrial biogenesis was also observed in ageing pathology.^35^, ^36^, ^37^, ^38^, ^39^, ^40^ Acupoint catgut embedding may ameliorate the age‐related decline in mitochondrial biogenesis (Figure 5A,H), which may partial compensate for dysfunction of mitochondrial during ageing.

## 5 CONCLUSION

In the present study, we demonstrated that treatment of acupoint catgut embedding on ageing rat could alleviate senescence with improving ageing‐related alteration in appearance, muscle function, spatial memory loss and reducing degenerated cells in the dentate gyrus, ameliorating mitochondrial dysfunction, and regulating ageing‐related decline of autophagic activity in the hippocampus. These effects were partially attenuated by the inhibition of autophagy. We also revealed that PINK1 other than PINK1/Parkin together involved in the regulation of mitophagy by acupoint catgut embedding, without mediating by p62. In addition, we observed no effect on imhomeostasis between mitochondrial fusion and fission by acupoint catgut embedding treatment. Furthermore, we discovered it could stimulate the increase of mitochondrial biogenesis, which may be compensative mechanism for dysfunction of mitochondria during ageing. However, this research was just a preliminary exploring for the effect of acupoint catgut embedding on ageing, which required much more complement and improvement on clarifying the exact molecular and interaction between them. Still, it offered a new trial for the inevitable ageing process, which may somehow release the heavy burden on public healthcare system.

## CONFLICTS OF INTEREST

We disclose no potential conflicts of interest.

## AUTHOR CONTRIBUTION

**Meiling Zhou:** Conceptualization (lead); Data curation (lead); Formal analysis (lead); Methodology (lead); Project administration (lead); Resources (lead); Software (lead); Validation (lead); Writing‐original draft (lead); Writing‐review & editing (lead). **Yumei Yuan:** Methodology (equal); Resources (equal). **Zishu Lin:** Methodology (equal); Supervision (equal). **Bin Zhang:** Investigation (equal); Project administration (equal); Supervision (equal). **Wenluo Qin:** Methodology (equal); Project administration (equal). **Yongqiang Liu:** Conceptualization (equal); Methodology (equal); Supervision (equal); Writing‐review & editing (equal). **Cong Yang:** Formal analysis (equal); Methodology (equal); Supervision (equal). **Qi Wang:** Conceptualization (equal); Resources (equal); Supervision (equal). **Hong Zhang:** Conceptualization (equal); Funding acquisition (lead); Investigation (equal); Project administration (lead); Resources (lead); Supervision (lead); Validation (equal).

## Data Availability

The data that support the findings will be available in Wiley at https://onlinelibrary.wiley.com/ following an embargo from the date of publication to allow for commercialization of research findings.
